# Carney triad: A case report, characteristics and literature review of this rare entity

**DOI:** 10.1016/j.ijscr.2020.12.054

**Published:** 2021-01-07

**Authors:** Iñaki Fraile Alonso, Rafael López Pardo

**Affiliations:** Department of General and Digestive Surgery, Hospital Virgen de la Salud, Avenida Barber nº30, Toledo, Spain

**Keywords:** Carney triad, Carney complex, Gastric tumor, Paraganglioma, Pulmonary condroma, Case report

## Abstract

**Introduction and importance:**

Carney triad is a rare entity of unknown etiology, characterized by the association of tumors with low incidence such as: gastric leiomyosarcoma, pulmonary chondroma and extra-adrenal paraganglioma. We show a case of Carney triad diagnosed in our center that has some different characteristics to the typical presentation of this patology, and a review of the literature.

**Case presentation:**

We present the case of a 47-year-old men who was admitted to our hospital for upper gastrointestinal bleeding and anemia. The patient was diagnosed of gastric GIST (gastrointestinal stromal tumor), pulmonary nodule and retroperitoneal nodule close to iliac bifurcation. A review of his medical history revealed that he had undergone surgery at 18-year-old for gastric leiomiosarcoma, subtotal gastrectomy performed with gastroenteric anastomosis type Billroth II.

The patient underwent total gastrectomy with a end-lateral esophago-jejune mechanical anastomosis and retroperitoneal tumor was also excised. Pathological result confirmed gastric GIST, and extra-adrenal paraganglioma.

**Clinical discussion:**

We review the available literature to determinate the characteristics and the most appropriate management for this syndrome. The trend is surgical treatment of gastric GIST and paraganglioma, preferred conservative treatment in pulmonary chondroma.

It is under discussion whether gastric tumors Carney syndrome is considered GIST, because the Carney triad lesions in stomach are different clinically, pathologically, and etiologically from GIST of stomach.

**Conclusion:**

Carney's triad is a low prevalence entity, with a limited number of publications, so the presentation of the syndrome may be different from the presentation described in the literature.

## Highlights

• Carney's syndrome gastric tumors differ in their characteristics from gastrointestinal stromal tumors (GIST).
• Carney syndrome could have different forms of presentation.
• The diagnosis of carney syndrome could occur up to 30 years after the diagnosis of the first tumor.

## 1 Introduction

Carney triad is a rare entity of unknown etiology, characterized by the association of tumors with low incidence such as: gastric leiomyosarcoma, pulmonary chondroma and extra-adrenal paraganglioma [[1], [2], [3], [4]]. We show a case of Carney triad diagnosed in our center that has some different characteristics to the typical presentation of this patology, and a review of the literature.

The work has been reported in line with the SCARE criteria.

Agha RA, Borrelli MR, Farwana R, Koshy K, Fowler A, Orgill DP, For the SCARE Group. The SCARE 2018 Statement: Updating Consensus Surgical CAse REport (SCARE) Guidelines, International Journal of Surgery 2018;60:132–136 [5].

## 2 Case presentation

We present the case of a 47-year-old man who was admitted to our hospital for upper gastrointestinal bleeding and anemia (hemoglobin 6.1 g/dl). His hemodynamic status was normal, but physical examination revealed pale conjunctiva and dry oral mucosa. Abdominal exploration without patology. A review of his medical history revealed that he had undergone surgery at 18-year-old for gastric leiomiosarcoma, subtotal gastrectomy performed with gastroenteric anastomosis type Billroth II. After follow-up was discharged. No drug history, no family history including any relevant genetic information, and psychosocial history.

**Fig. 1:**
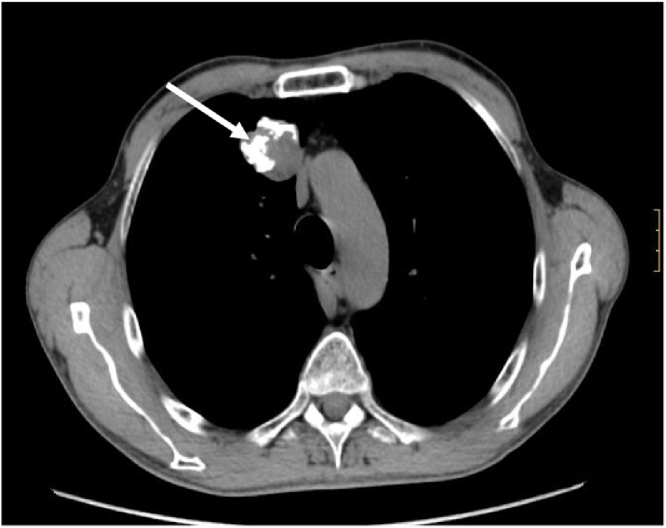
TC shows calcified image compatible with lung hamartoma (white arrow).

**Fig. 2:**
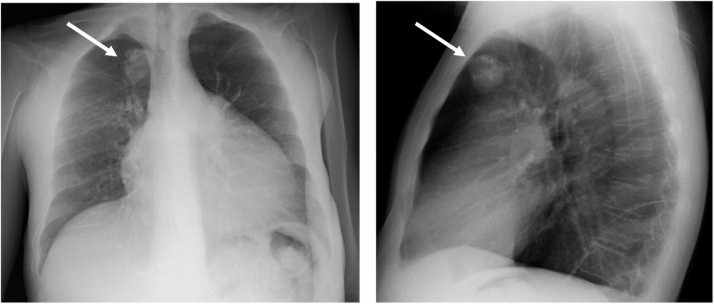
The chest radiograph showed one round calcified tumor (white arrow) in the right lung.
• Gastric tumor of 13.5cm × 7.5cm with soft tissue density, extending into pancreas, without infiltration (Fig. 3).

Gastroendoscopic revealed a tumor over greater curvature extending into posterior wall, of about 5 cm of diameter, soft consistency and very friable. Biopsies are reported as gastrointestinal stromal tumor (GIST). A toracoabdominopelvic computerized tomography (CT) showed:

• Lung nodule located in upper right lobe with calcifications (Fig. 1, Fig. 2).

**Fig. 3:**
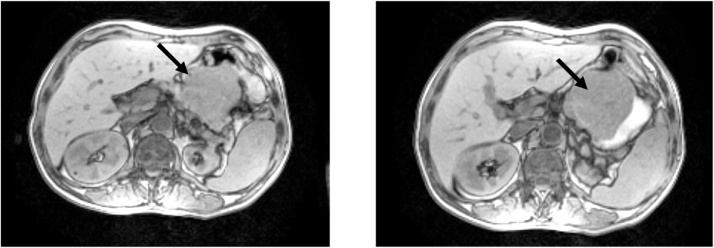
Abdominal NMR shows a large mass that seems to depend on the stomach with no clear separation plane with the pancreas. The black arrow indicates the gastric mass.
• Retroperitoneal nodule close to iliac bifurcation.

Positron Emission Tomography (PET) was performed showing pathological uptake in stomach with radiological characteristics suggestive of malignancy.

**Fig. 4:**
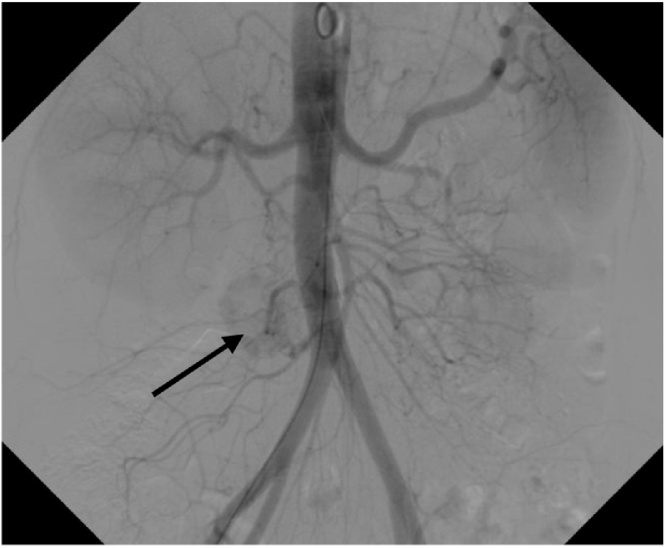
Arteriography. Hypervascular nodule seems to depend on lumbar artery. Black arrow indicates hypervascular nodule.

This diagnosis prompted further study with nuclear magnetic resonance (NMR). Imaging evidenced a gastric tumor without clear separation plane with pancreas. Study is completed by arteriography, showing a hypervascular retroperitoneal nodule (Fig. 4). The urinary content of metanephrines/catecholamines was negative.

This is a patient with an initial suspicion of a gastric tumor who in the extension study is diagnosed with two tumors at different levels (pulmonary and retroperitoneal), which makes us suspect the possibility of a multisystemic syndrome.

Surgical management was decided after overall clinical condition improved (transfusion and enteral protein supplements). The patient was informed of the risks of the surgery and of the possible sequelae, the patient accepting to the proposed surgical treatment.

The procedure was performed by a surgery specialist in esophagogastric surgery. General anesthesia. Suprainfraumbilical median laparotomy. Abdominal approach revealed a gastric tumor of 15 cm in the gastroenteric anastomosis, liver mass in segment III and a retroperitoneal nodule 4 cm above the inferior cava vein was also observed. The patient underwent total gastrectomy, preserving pancreas and spleen, with a end-lateral esophago-jejune mechanical anastomosis. Retroperitoneal tumor were also excised.

The patient had uneventful recovery. No had postoperative complications. The patient was discharged on the tenth postoperative day with dietary recommendations and control in consultations without presenting complications after discharge. The patient has undergone surgical and oncology controls without showing signs of recurrence.

The pathological result shown: gastrointestinal stromal tumor (GIST), positive for c-kit, CD 34, and negative for vimentin, actin and desmin in inmunohistochemical studies, with low proliferation index. Analysis of the liver specimen showed liver hemangioma and the retroperitoneal tumor was diagnosed of paraganglioma.

After nine years of follow-up it has not been demonstrated tumor recurrence. The pulmonary lesion has not undergone significant changes.

## 3 Discussion

Carney triad was firstly described by Carney, pathologist of the Mayo Clinic in 1977, consisting of: the association of three rare tumors: gastric leiomyosarcoma, pulmonary chondroma and extra-adrenal paraganglioma, [[1], [2], [3],^6^]. Subsequently other tumors were added to the syndrome as, esophageal leiomyoma, and adrenal cortical adenoma [3,4].

The diagnosis of the original triad is not the usual presentation of this syndrome, found in only 22% of the patients [2,6,7]. The case we report presents the peculiarity that was initially diagnosed with gastric leiomyosarcoma as an isolated entity, at 18 years old, with no tumor recurrence at follow up. Twenty-nine years later the patient was a diagnosis of recurrence gastric tumor, lung lesion and retroperitoneal nodule. After surgery and histological examination, the patient was diagnosed of Carney triad (has not been obtained pathological confirmation of lung lesion is chondroma, but the radiological characteristics and stability of the lesion suggest pulmonary chondroma as first possibility diagnostic).

This case did report not show typical manifestations in this syndrome. The disorder affects mostly young women, around 80% cases are women [3,4], whereas our patient was a men of 47 years-old. As previously mentioned, the original triad is not the common presentation of this syndrome, (usual presentation is the combination of gastric and lung injuries), thus the syndrome is usually only partially expressed [3]. The presence of at least two of these three rare tumors is considered sufficient for diagnosing the syndrome [[6], [7], [8]]. Our patient presented the three tumors of Carney triad, with the peculiarity that 29 years earlier he had been diagnosed of gastric leiomyosarcoma. It is possible that the interval between tumors presentation will be long, hence the importance in these patients of long term follow up [3,9].

The presentation of this patology may be varied, since an asintomatic patient with indicental discover in a radiography, until ferropenic anemia caused by bleeding of gastric tumor or even malignant hypertension crisis by the cathecolamine secretion of a paraganglioma [3,4,6,7,10]. The most common initial clinical manifestation is upper gastrointestinal bleeding and associated symptoms and signs (e.g. anemia, hematemesis, and melena) [3,7,10].

### 3.1 GIST or not GIST

Gastric tumors is the most commonly observed component of Carney triad (99%) [2,6]. Several titles were given to these gastric tumors, initially most intestinal intramural mesenchymal spindle cell tumors were thought to arise in smooth muscle and, consequently, were interpreted as leiomyomas or leiomyosarcomas. After in 1987 these gastric tumors were shown to arise from the interstitial cells of Cajal [11] so they were included under gastrointestinal stromal tumors (GIST).

Gastrointestinal stromal tumors (GISTs) are the most common mesenchymal neoplasms of the gastrointestinal tract. They harbor specific activating mutations in the KIT (KIT positive (CD117)) or platelet-derived growth factor receptor a (PDGFRA) receptor tyrosine kinases, which makes them responsive to pharmacologic inhibitors, such as imatinib mesylate and sunitinib malate [12,13]. However, because the Carney triad lesions are different clinically, pathologically, behaviorally, and etiologically from gastrointestinal stromal tumors of stomach (Table 1), so gastric stromal sarcoma is a more accurate terminology. Our case was firstly diagnosed with leiomyosarcoma and the second surgery with GIST, showing the evolution of classifications that have been occurring in gastric tumors of Carney triad.

**Table 1:** Comparison characteristics sporadic gastric GIST vs Gastric stromal sarcoma in Carney triad.

Feature	GIST	Gastric stromal sarcoma in Carney Triad
**Cause**	Mutation somatic KIT or PDGFRA	UnKnown
**Age**	Mean 50 years old	Mean 20 years old
**Sex ratio**	Similar	Female predilection
**Location**	Body	Antrum
**Imatinib, sunitinib treatment**	Good response	Poor response

The most common initial clinical manifestation is upper gastrointestinal bleeding and associated symptoms and signs (e.g. anemia, hematemesis, and melena) abdominal pain is associated sometimes [6,10]. Asymptomatic abdominal mass discovered in the study of other pathology also could be a clinical presentation.

The metastatic spread in these tumors is common and early (55% of cases have metastases at diagnosis), especially in the lymph nodes, liver and peritoneum. These metastases usually do not produce symptoms [3].

The gastric sarcomas were treated surgically with procedures ranging from local tumor excision to total gastrectomy. Experience has shown that early gastric resection, with removal of as much of the organ as is consistent with preservation of function, is the treatment of choice for gastric sarcomas [3,6,7]. These tumors have a tendency to recidive in the gastric remnant so it is sometimes necessary to perform a new surgery and total gastrectomy [3]. Metastases of these tumors were unresponsive to chemotherapy, radiotherapy, and hyperthermia. In particular, targeted therapy (imatinib and sunitinib) that is successful in treatment of sporadic GIST was ineffective, or had unclear positive results [3,6].

### 3.2 Extra-adrenal paraganglioma

Paragangliomas are rare tumors, 10–15% has extraadrenal presentation, and being the extra-adrenal paraganglioma the less common component of Carney triad (was reported in only 47% of patients). Paragangliomas are divided into functioning (can produce hypertensive crisis, tachycardia, palpitations), and no functioning that usually have no symptoms, with casual diagnosis in autopsy of regional surgery. If symptoms are present, this derivated the location, tumor growth and expansion, the most common symptom is pain and the presence of an abdominal mass [3,6].

The paraganglioma should be resected if it is accessible owing to the possibility of metastasis, by growth that could compromise vascular and neural structures and the positive outcomes of curative surgery [3,6]. Has been described selective embolization for the treatment of extra-adrenal paraganglioma [14], however, more studies are needed to clarify its role in managing the Carney triad.

### 3.3 Pulmonary chondroma

Pulmonary chondromas are benign tumors present in 76% cases of Carney syndrome [3], are mostly cases multiple and bilateral. Histologically they are composed mainly of cartilage, differing from hamartomas where bronchial epithelium and cartilage are present. The lesions were usually discover incidentally in radiographs to study other patology or control during follow-up for gastric neoplasms. CT scan is a useful tool to detect thin peripheral calcification in the chondromas that may not be evident on a standard radiograph, but the definitive diagnosis requires a biopsy [9,15]. These tumors are usually not present clinical, therefore in mostly cases it is not necessary treatment, only follow up.

In those rare cases that the chondroma could cause respiratory symptoms such as dyspnea, atelectasis, recurrent pulmonary infections or hemoptysis a tumor resection by thoracotomy or thoracoscopy could be performed [3,6,15].

## 4 Conclusion

Carney triad has a low incidence, with a limited number of relevant publications, so the presentation of the syndrome may be different from the presentation described in the literature.

Carney's syndrome gastric tumors differ in their characteristics from gastrointestinal stromal tumors (GIST).

Treatment involves surgical removal of some components of the triad (gastric tumors, paraganglioma), and the need of long term follow up for possible recurrence. Pulmonary condroma do not need to be excised unless they are symptomatic.

## Declaration of Competing Interest

No conflicts of interest.

## Funding

No sources of funding.

## Ethical approval

The study is exempt from ethical approval in my institution.

## Consent

Authors have written and signed consent to publish a case report from the patient.

## Registration of research studies

Not Applicable.

## Guarantor

Iñaki Fraile Alonso.

## Provenance and peer review

Not commissioned, externally peer-reviewed.

## CRediT authorship contribution statement

**Iñaki Fraile Alonso:** Conceptualization, Formal analysis, Investigation, Methodology. **Rafael López Pardo:** Supervision, Validation.
